# Supplementary material for: Histopathologic image–based deep learning classifier for predicting platinum-based treatment responses in high-grade serous ovarian cancer
Source: Nat Commun. 2024 May 18;15:4253. doi: 10.1038/s41467-024-48667-6 (PMC11102549; doi:10.1038/s41467-024-48667-6)
Supplement: Supplementary file 1 — Supplementary Information [file 41467_2024_48667_MOESM1_ESM.pdf]

Supplementary Data

Histopathologic Image–based Deep Learning Classifier for Predicting Platinum-based Treatment Responses in High-grade Serous Ovarian Cancer

Byungsoo Ahn, Damin Moon, Hyun-Soo Kim, Chung Lee, Nam Hoon Cho, Heung-Kook Choi, Dongmin Kim, Jung-Yun Lee, Eun Ji Nam, Dongju Won, Hee Jung An, Sun Young Kwon, Su-Jin Shin, Hye Ra Jung, Dohee Kwon, Heejung Park, Milim Kim, Yoon Jin Cha, Hyunjin Park, Yangkyu Lee, Songmi Noh, Yong-Moon Lee, Sung-Eun Choi, Ji Min Kim, Sun Hee Sung, Eunhyang Park*

Corresponding author E-mail address: epark54@yuhs.ac

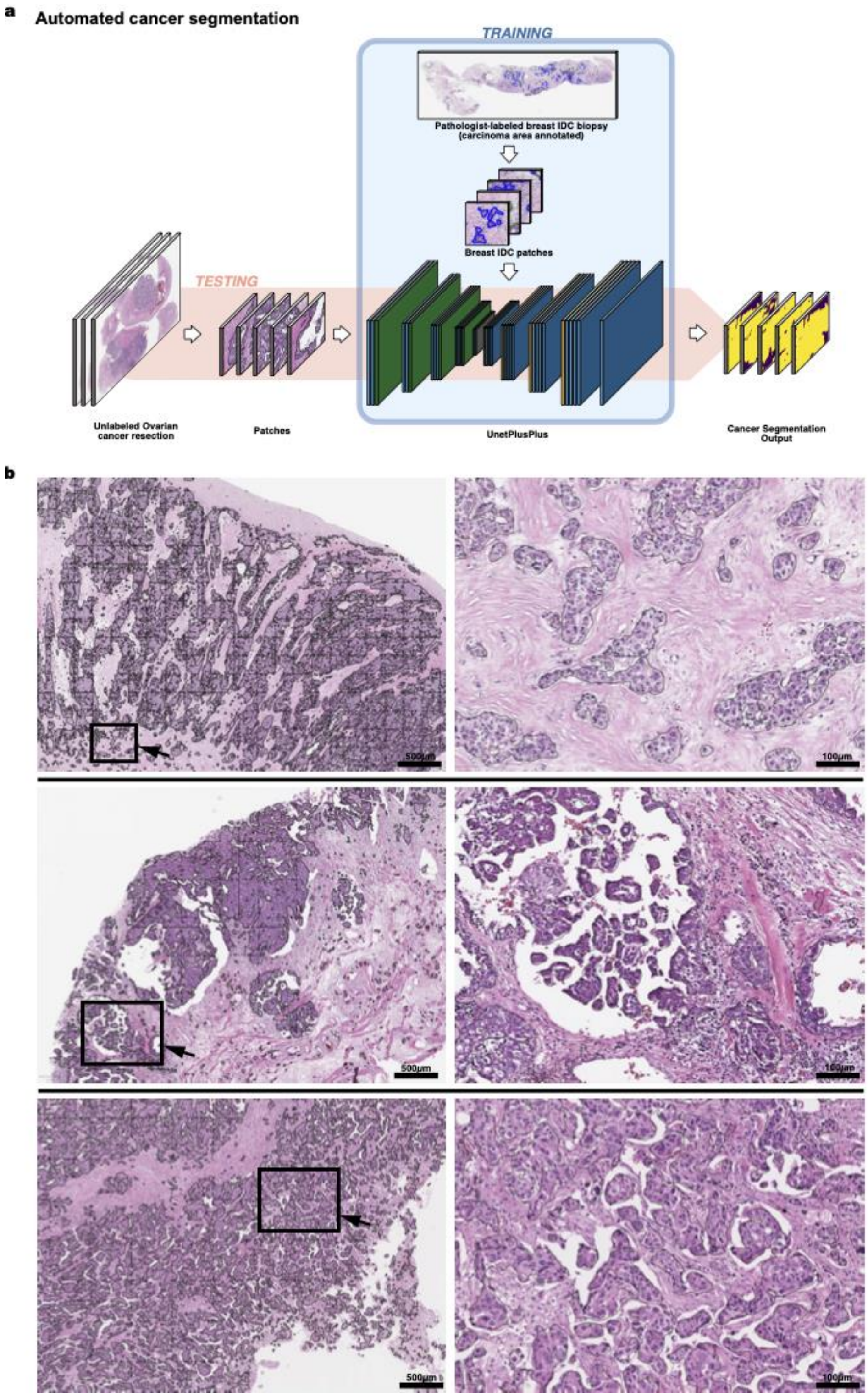

Supplementary Figure 1. Automated cancer segmentation model.

a, For the cancer-segmented area, the UNetPlusPlus model, which had been pre-trained on pathologist-labeled breast invasive ductal carcinoma for cancer segmentation, was used to label patches from the ovarian cancer resection whole slide images. **b**, Representative images of automated cancer segmentation (left: low-magnification, right: high-magnification).

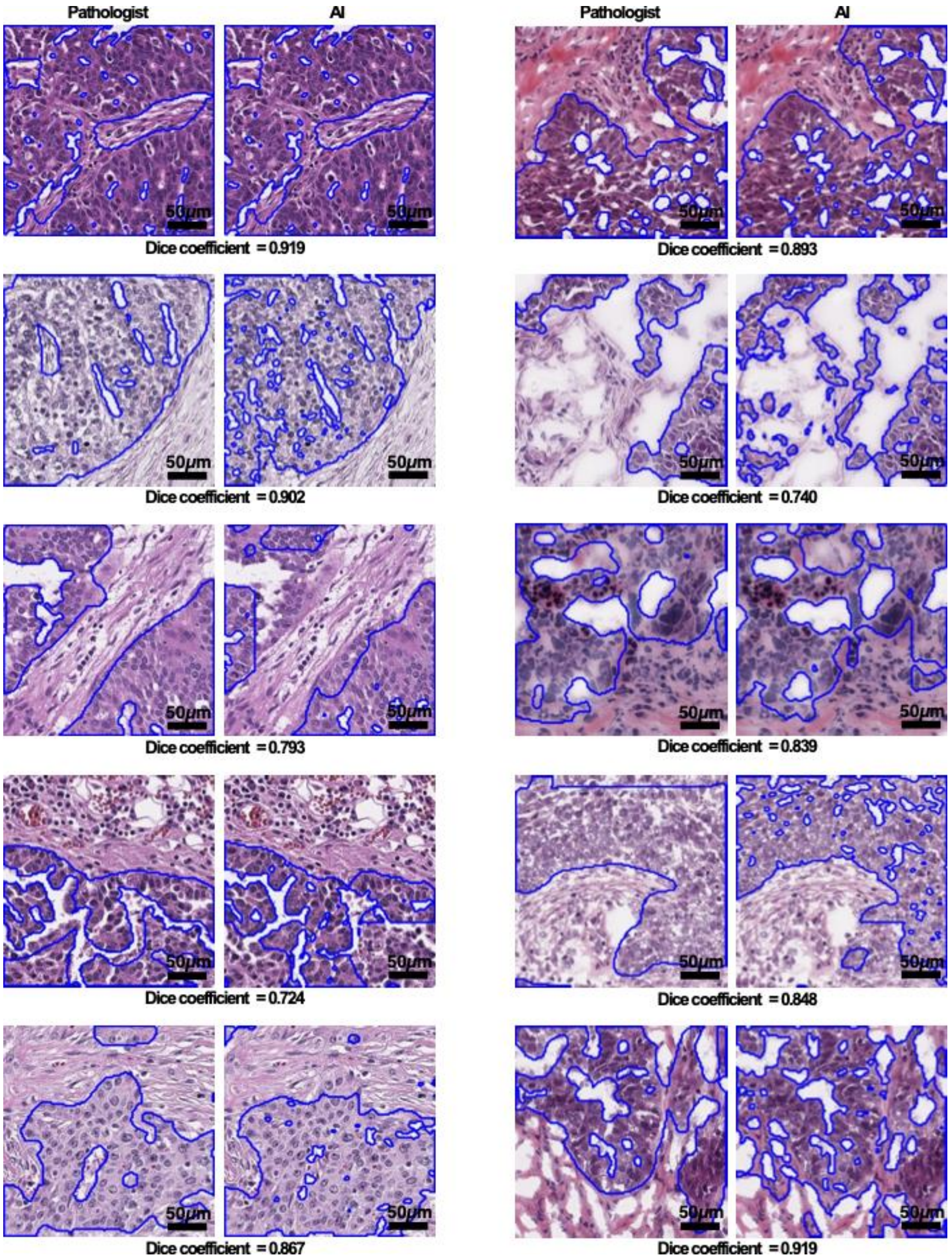

Supplementary Figure 2. Automated cancer segmentation model validation with concordance analysis between the pathologist-annotated area and the model's cancer segmented area.

10% of the cases from the internal (SEV) and external (TCGA) cohorts were randomly selected, and 250 x 250 µm regions from the whole slide images were manually annotated by experienced gynecologic pathologists. Side-by-side comparisons between the pathologists' and automated cancer segmentation model's areas are shown with the corresponding Dice coefficients.

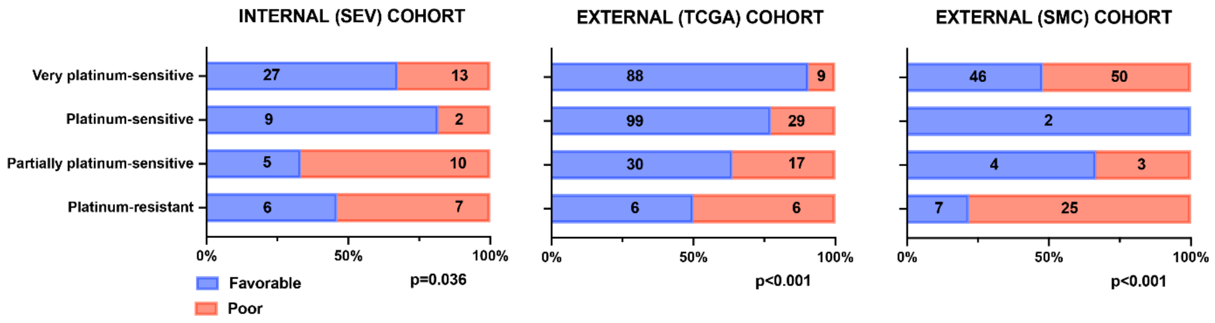

Supplementary Figure 3. Distribution of PathoRiCH prediction results across three different cohorts: the internal (SEV) and external (TCGA and SMC).

The distribution of PathoRiCH prediction outcomes for three patient cohorts–internal (SEV) and external (TCGA and SMC) cohorts, broken down into four ground truth PFI groups: platinum resistant (PFI ≤6 months), partially platinum resistant (6–12 months), platinum sensitive (12–24 months), and very platinum sensitive (>24 months). The colored bars indicate the percentage of predictions for each outcome group (blue for favorable and red for poor), with numerical values within each bars showing the case count for each category. The predictions for all three cohorts showed significantly different distributions for the four PFI groups (p = 0.035, p < 0.001 and p < 0.001 respectively).

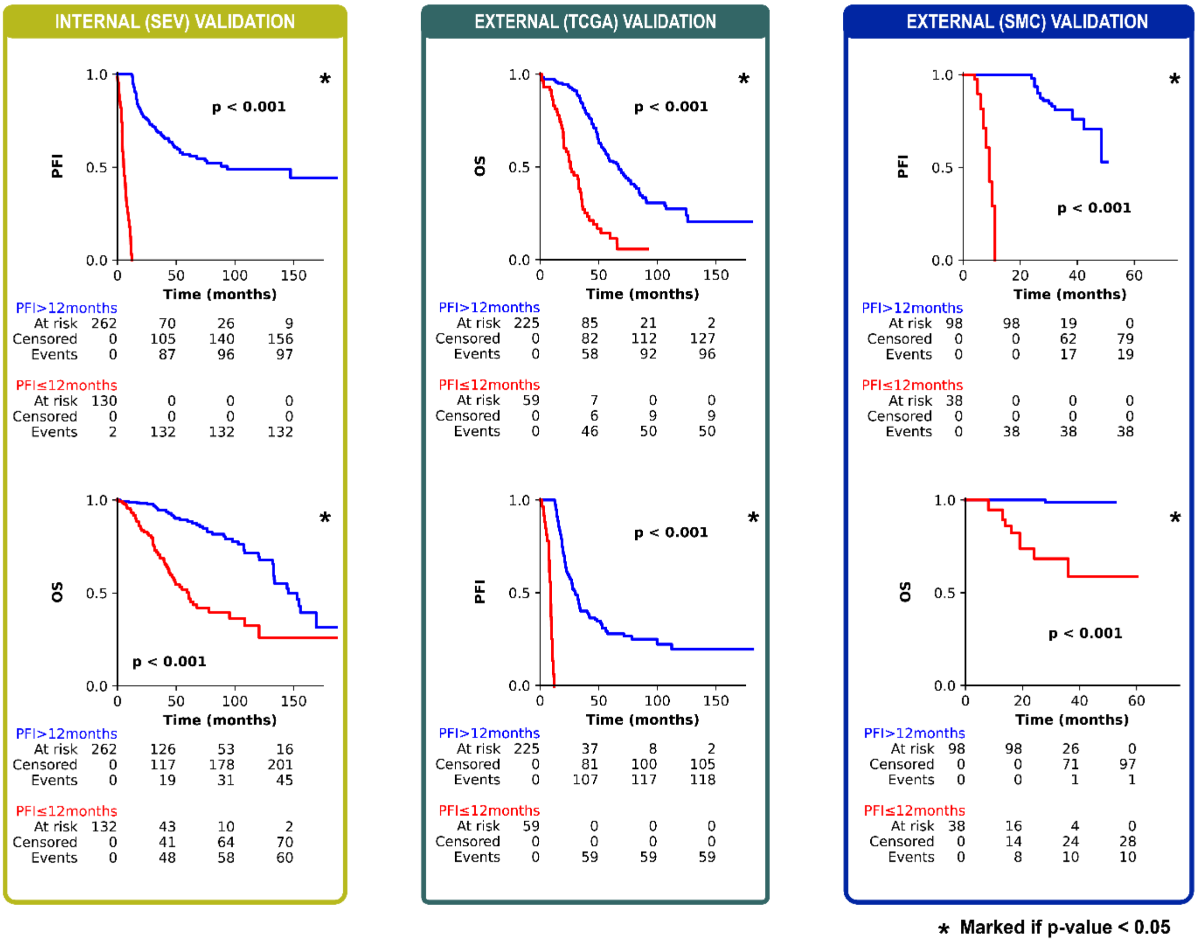

Supplementary Figure 4. Kaplan-Meier survival analysis plots depicting ground truth favorable and poor groups in three different cohorts: the internal (SEV) and external (TCGA and SMC).

All three cohorts displayed statistically significant patient stratification for both the platinum-free interval (PFI) and overall survival (OS) ($p < 0.001$ for all analyses).

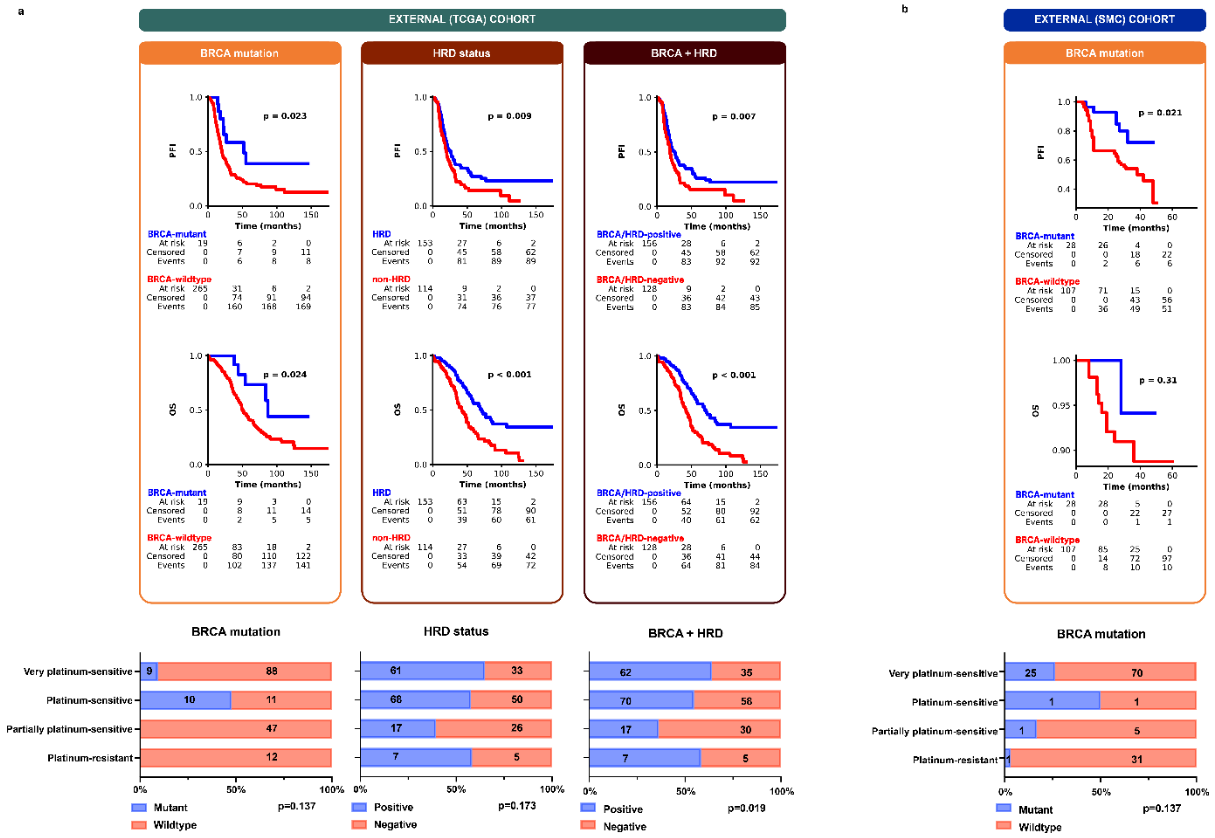

Supplementary Figure 5. The Kaplan-Meier plots and distributions of the four PFI groups according to *BRCA* mutation, HRD status, and combined *BRCA* and HRD status in the external (TCGA and SMC) cohorts.

a, Kaplan-Meier survival plots of external TCGA cohort patients categorized by their *BRCA* mutation status, HRD status, and combined *BRCA* and HRD status. All factors demonstrated significant stratification for favorable and poor prognostic groups in terms of PFI (p = 0.023, p = 0.009, and p = 0.007, respectively) and OS (p = 0.024, p < 0.001, and p < 0.001, respectively). Accompanying bar graphs below depicted the percentage of patients across four PFI groups within the TCGA cohort, stratified by *BRCA* mutation, HRD status, and combined *BRCA* and HRD status. A statistical difference in distribution was only seen in combined *BRCA* and HRD status (p = 0.019).

b, The external SMC cohort's survival analysis showed significant stratification based on *BRCA* mutation status for PFI (p = 0.021), but not for OS (p = 0.31). The associated bar graph showed the patient distribution across four PFI categories based on *BRCA* mutation status, which did not provide a significant distinction between the groups (p = 0.137).

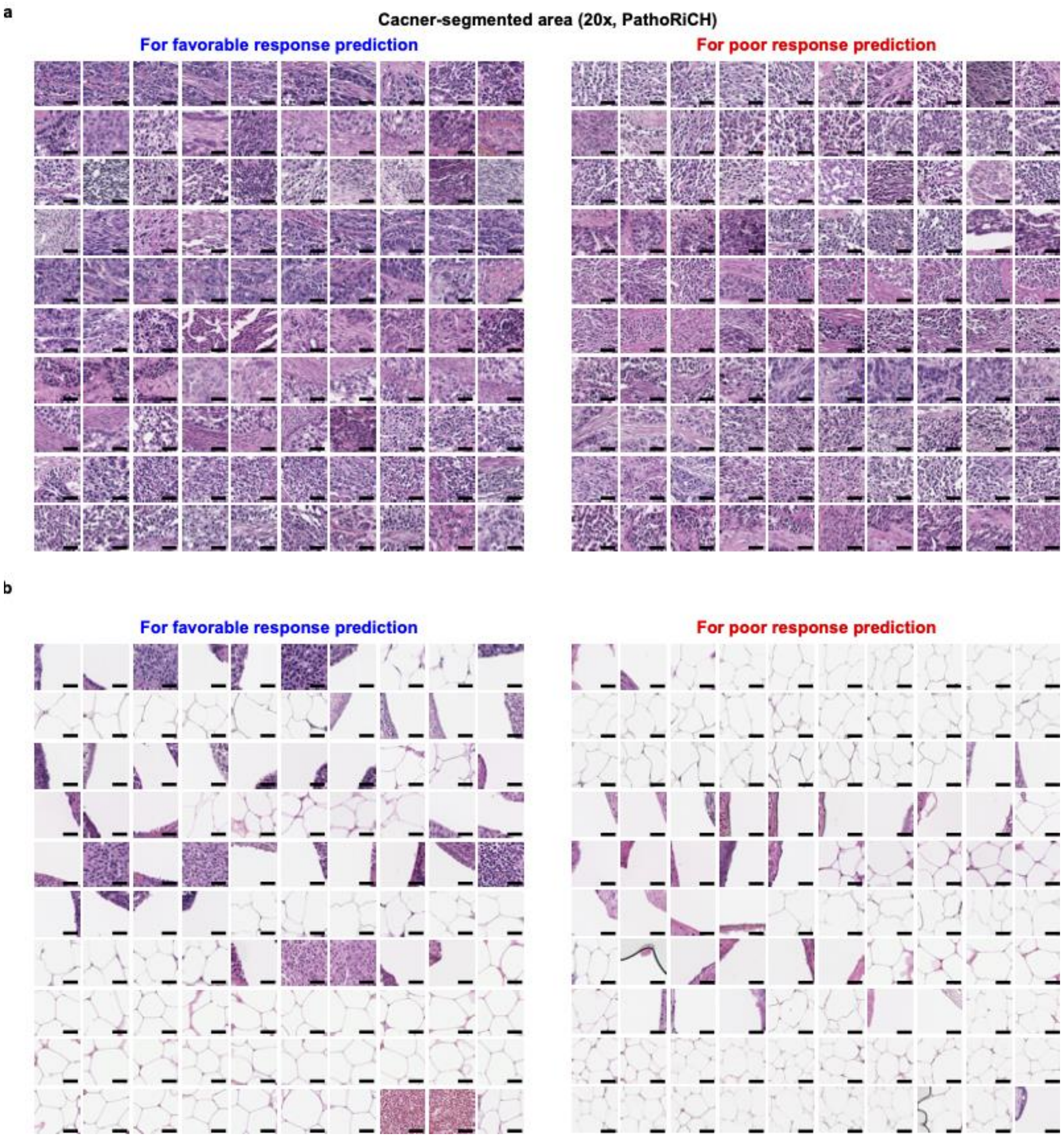

Supplementary Figure 6. The top 100 patches for the favorable and poor response groups predicted by PathoRiCH and the all-tissue area 20× magnification model. (Scale bar = 50µm for all patch images) **a.** Top 100 patches for the PathoRiCH-predicted favorable and poor response groups. **b.** Top 100 patches for the favorable and poor response groups determined by the all-tissue area 20× magnification model.

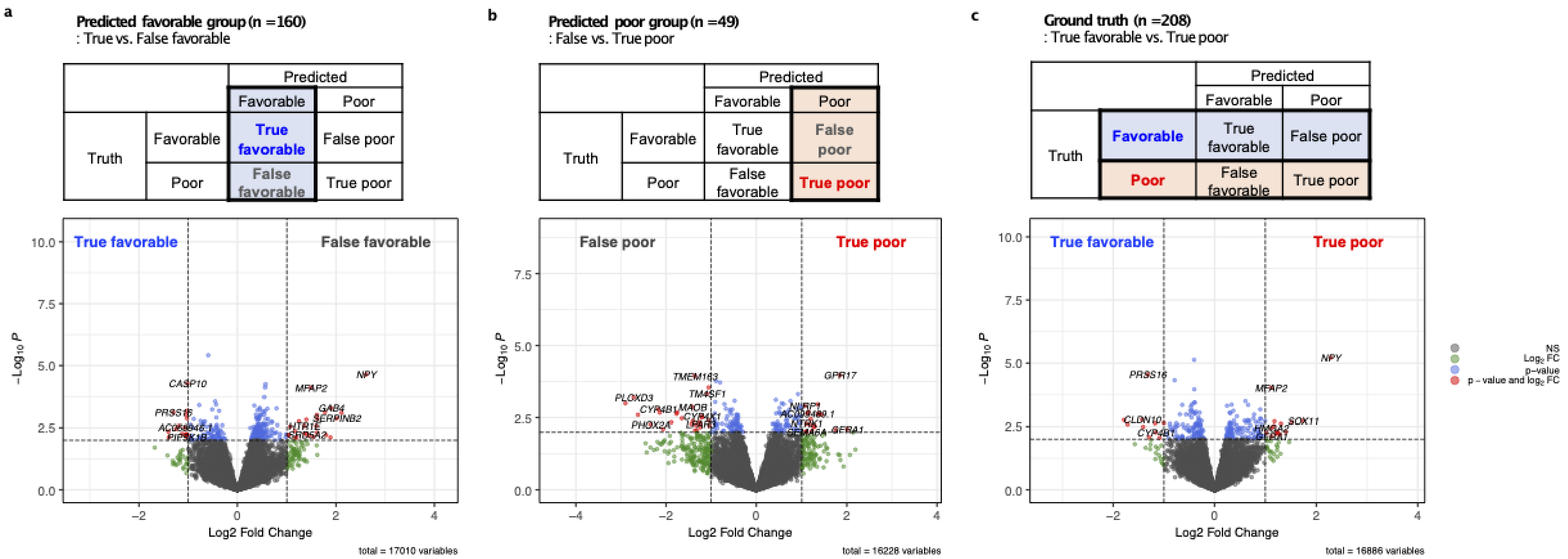

Supplementary Figure 7. Volcano plots of the PathoRiCH-predicted and actual ground truth response groups.

a. Favorable-predicted group, contrasting between true and false predictions. **b.** Poor-predicted group, showing expression differences between false and true predictions. **c.** Ground truth data, distinguishing true favorable and true poor groups. Horizontal dotted line: cut-off of $p < 0.01$, vertical dotted line: cut-off of absolute log2 fold change > 1.

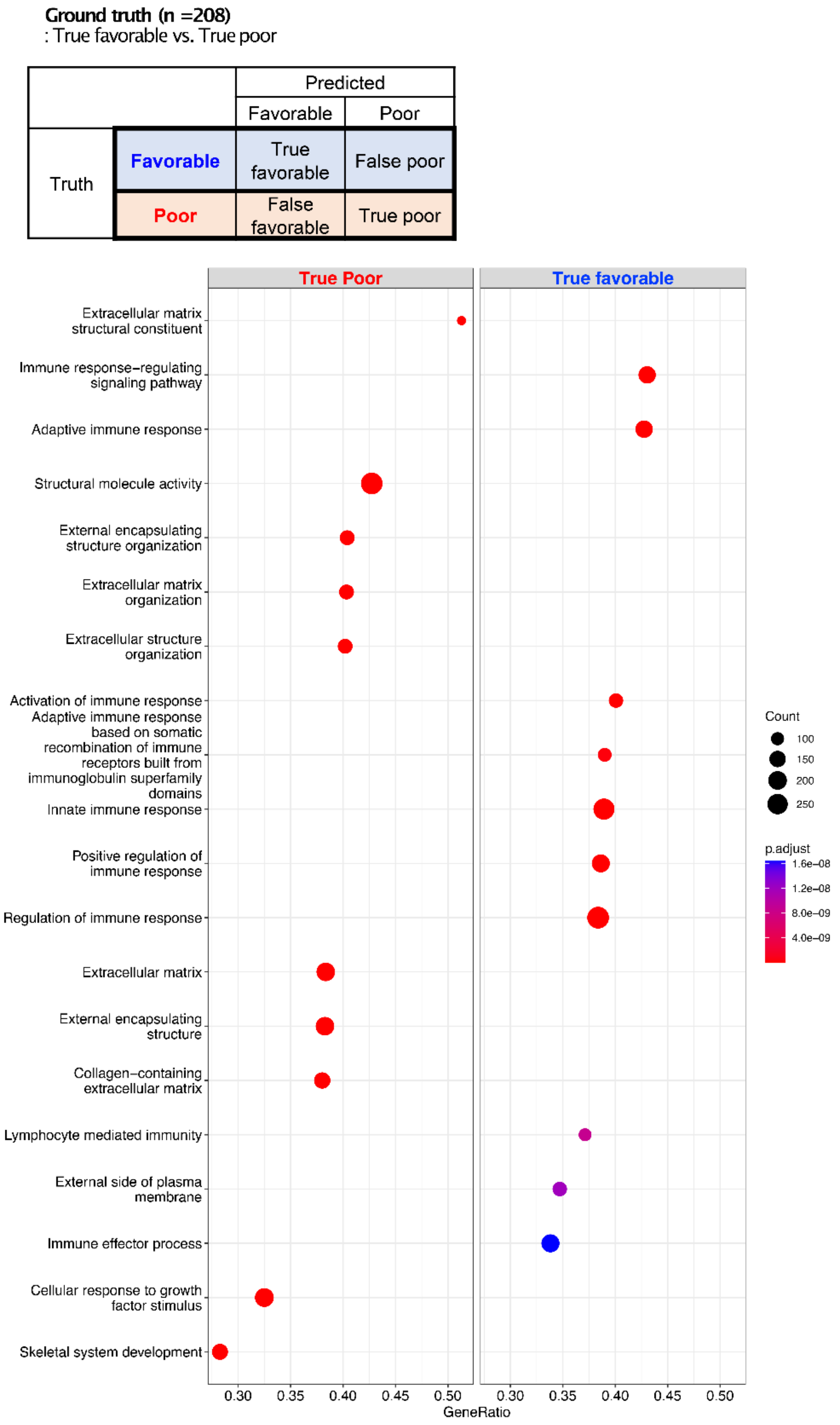

Supplementary Figure 8. Differential gene ontology (GO) profiles comparing the true favorable-response and true poor-response groups.

The confusion matrix highlights the true favorable and true poor groups. The GO biological process analysis reveals that the true favorable group is predominantly enriched in immune response–related genes, whereas the true poor group predominantly features genes involved

in extracellular matrix–associated processes. ClusterProfiler[1] with a Benjamini–Hochberg procedure was used for GO analysis.

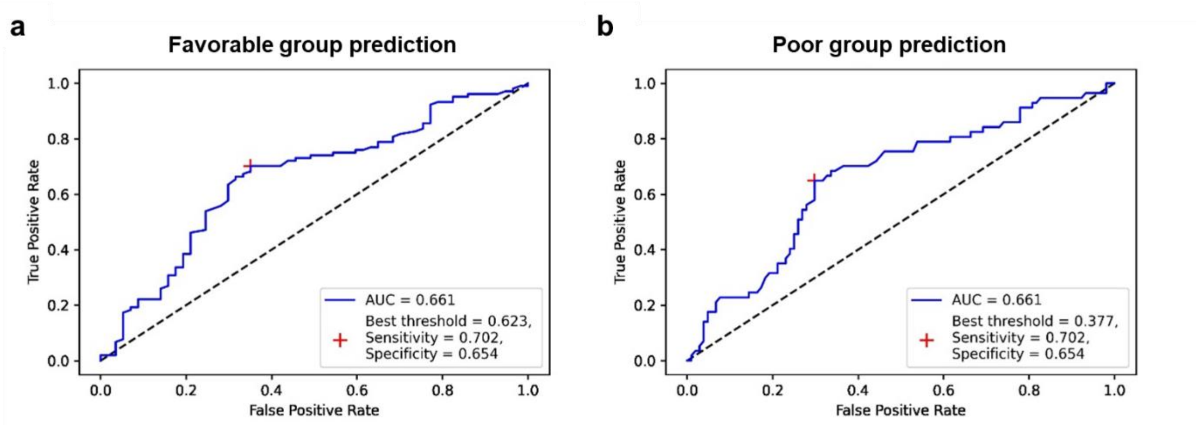

Supplementary Figure 9. Receiver operating characteristic curves of the Youden's Index for identifying the thresholds for favorable and poor response groups

a, For predicting favorable response group, 0.623 was identified as the optimal threshold. **b**, For predicting poor response group, 0.377 was identified as the optimal threshold.

Supplementary Table 1. Cancer segmentation concordance between pathologists and cancer segmentation model

	Dice coefficient (Mean)
SEV cohort (n=39)	0.781
TCGA cohort (n=29)	0.836
Overall	0.804

Supplementary Table 2. Performance of cancer-segmented area multiple instance learning models and ensemble analyses for the internal (SEV) and external (TCGA) validation cohorts

		Cancer-segmented area					
		5×	20×	Multiscale	Soft voting (5×, 20×)	Hard voting (AND) (5×, 20×)	Hard voting (OR) (5×, 20×)
Internal validation (SEV cohort)	AUC-ROC*	0.604 ± 0.05	0.596 ± 0.072	0.614 ± 0.046	0.586	0.635	0.587
	Precision	0.521	0.465	0.507	0.424	0.465	0.518
	Recall	0.468	0.675	0.525	0.562	0.739	0.374
	F1 Score	0.470	0.522	0.507	0.483	0.566	0.418
	K-M *p* value**	0.000	0.000	0.000	0.059	0.011	0.149
External validation (TCGA cohort)	AUC-ROC	0.532	0.602	0.573	0.553	0.579	0.578
	Precision	0.519	0.406	0.407	0.500	0.398	0.556
	Recall	0.250	0.528	0.481	0.245	0.538	0.250
	F1 Score	0.338	0.459	0.441	0.329	0.457	0.345
	K-M *p* value**	0.000	0.032	0.036	0.000	0.023	0.000

AUC-ROC, area under the receiver operating characteristic curve; K-M, Kaplan-Meier analysis (two sided)

*From 5-fold cross validation; **Based on platinum-free interval

Supplementary Table 3. Clinical and molecular characteristics of the groups predicted to respond to platinum-based treatment in the TCGA and SMC external validation cohorts

	TCGA cohort			SMC cohort		
	Favorable (N=223)	Poor (N=61)	*p*	Favorable (N=59)	Poor (N=77)	*p*
Age	59.6 ± 11.3	60.6 ± 10.8	0.555	56.3 ± 8.9	57.4 ± 8.7	0.505
FIGO Stage			7.82E-04			0.646
Stage I	11 (5.0%)	1 (1.6%)		7 (11.9%)	5 (6.5%)	
Stage II	22 (9.9%)	2 (3.3%)		5 (8.5%)	9 (11.7%)	
Stage III	182 (82.0%)	45 (73.8%)		34 (57.6%)	43 (55.8%)	
Stage IV	7 (3.2%)	13 (21.3%)		13 (22.0%)	20 (26.0%)	
BRCA mutation status			0.737			0.163
Mutant	16 (7.2%)	3 (4.9%)		16 (27.1%)	12 (15.8%)	
Wildtype	207 (92.8%)	58 (95.1%)		43 (72.9%)	64 (84.2%)	
HRD status (Telli et al.)[2]			0.289			
Positive	126 (59.2%)	27 (50.0%)				
Negative	87 (40.8%)	27 (50.0%)				
HRD status (Takaya et al.)[3]			0.976			
Positive	112 (50.5%)	28 (49.1%)				
Negative	110 (49.5%)	29 (50.9%)				
HRD status (Perez-Villatoro et al.)[4]			0.66			
Positive	56 (83.6%)	12 (75.0%)				
Negative	11 (16.4%)	4 (25.0%)				

HRD, homologous recombination deficiency
Two-sided unpaired t-tests were used for analysis

Supplementary Table 4. Univariate and multivariate survival analyses for the platinum-free interval of patients with high-grade serous ovarian carcinoma in the TCGA and SMC cohorts

	Univariate				Multivariate			
Variables	HR	95% CI	z	*p*	HR	95% CI	z	*p*
TCGA cohort								
Age (<60 vs. ≥60)	1.148	0.854–1.543	0.914	0.361	1.08465	0.796–1.478	0.515	0.607
Stage (I–II vs. III–IV)	2.676	1.488–4.812	3.289	0.001*	2.32104	1.281–4.204	2.778	0.005*
BRCA (Mutant vs. Wild)	2.236	1.098–4.551	2.218	0.026*	2.70652	1.092–6.707	2.15	0.032*
HRD (Positive vs. Negative)	1.497	1.102–2.034	2.58	0.010*	3.16161	0.748–13.359	1.566	0.117
BRCA+HRD (Positive vs. Negative)	1.489	1.096–2.022	2.544	0.011*	0.38607	0.089–1.673	-1.272	0.203
PathoRiCH (Favorable vs. Poor)	2.447	1.733–3.456	5.084	3.70E-07**	2.04982	1.441–2.916	3.991	6.57E-05**
SMC cohort								
Age (<60 vs. ≥60)	1.038	0.6133–1.758	0.14	0.889	0.86	0.5047–1.466	-0.554	0.579
FIGO Stage (I–II vs. III–IV)	6.018	1.871–19.36	3.011	0.003*	5.6049	1.7367–18.088	2.883	0.004*
BRCA (Mutant vs. Wild)	2.625	1.126–6.122	2.234	0.026*	2.2033	0.9414–5.157	1.821	0.069
PathoRiCH (Favorable vs. Poor)	1.885	1.074–3.307	2.208	0.027*	1.8233	1.0317–3.222	2.067	0.038*

CI, confidence interval; HR, hazard ratio; HRD, homologous recombination deficiency

Two-sided unpaired t-tests, Mann–Whitney U tests, and Wilcoxon rank-sum tests were employed for analysis

* p <0.05; ** p <0.0001

Supplementary Table 5. Clustering analyses of high-scoring patches for the PathoRiCH-predicted favorable and poor response groups

Clusters	Patch proportion (N of patches)	
	Favorable	Poor
F1 (favorable patches >90%)	90.5% (1268/1401)	9.5% (133/1401)
F2 (favorable patches 80%–90%)	83.9% (397/473)	16.1% (76/473)
M1 (mixed patches)	76.1% (648/852)	23.9% (204/852)
M2 (mixed patches)	66.9% (445/665)	33.1% (220/665)
M3 (mixed patches)	60.4% (297/492)	39.6% (195/492)
P2 (poor patches 80%–90%)	19.5% (176/903)	80.5% (727/903)
P1 (poor patches >90%)	9.8% (166/1687)	90.2% (1521/1687)

N, number

Supplementary Table 6. Correlation of the ground truth and predicted platinum-treatment response groups in the external (TCGA) cohort with available RNAseq results

		Predicted		
		Favorable	Poor	Total
Ground truth	Favorable	True favorable-predicted 134 (84.4%)	False poor-predicted 30 (61.2%)	164
Ground truth	Poor	False favorable-predicted 25 (15.6%)	True poor-predicted 19 (38.8%)	44
Total		159	49	208

Supplementary Table 7. Performance of multiple-instance learning models in predicting *BRCA* mutations

	BRCA mutation status<br>Cancer-segmented area MIL		
	5×	20×	Multiscale
AUC-ROC	0.525	0.526	0.456
Precision	0.934	0.949	0.944
Recall	0.598	0.349	0.036
F1 Score	0.731	0.511	0.069

AUC-ROC, area under the receiver operating characteristic curve; MIL, multiple instance learning

Supplementary Table 8. Performance of multiple-instance learning models in predicting homologous recombination deficiency

	HRD status (TCGA 8:2 split for training and test)								
	HRD status (Telli et al.[2])			HRD status (Takaya et al.[3])			HRD status (Perez et al.[4])		
	5×	20×	Multiscale	5×	20×	Multiscale	5×	20×	Multiscale
AUC-ROC	0.524	0.484	0.451	0.469	0.556	0.514	0.357	0.171	0.407
Precision	0.9	0.742	0.648	0.714	0.75	0.586	1	1	0.871
Recall	0.148	0.383	0.968	0.189	0.226	0.32	0.036	0.036	0.964
F1 Score	0.254	0.505	0.776	0.299	0.348	0.415	0.069	0.069	0.915

AUC-ROC, area under the receiver operating characteristic curve; HRD, homologous recombination deficiency

Supplementary Table 9. Previously published studies on histology-based prediction models for high-grade serous ovarian carcinoma prognosis using machine/deep learning

Publish date (Author)	Model	Training cohort	Internal validation cohort	External validation cohort	Performance	Prediction	Limitation
2020 (Yu *et al.*) [5]	VGGNet CNN architecture pretrained with ImageNet dataset	Patient n = 221<br>WSI n = unknown<br>(TCGA)	Patient n = 56<br>WSI n = unknown<br>(with 5-fold cross-validation)<br>(TCGA)	Not done	p = 0.003*	Platinum-free interval (PFI) prediction (regression)	- Included all serous ovarian adenocarcinoma (including low-grade serous carcinoma)<br>- No external validation
2021 (Laury *et al.*)[6]	Weakly supervised CNN with multiple label-revision steps by pathologists<br>1. Weakly supervised tumor segmentation CNN using patient outcome<br>2. Supervised learning based on hard labels (the output of weakly supervised tumor segmentation CNN) for digital biomarkers	Patient n = 30<br>WSI n = 205<br>(single institution)	Patient n = 22<br>WSI n = 22<br>(Single institution)	Not done	Sensitivity: 73%<br>Specificity: 91%<br>PPV: 89%<br>Overall accuracy: 82%	PFI prediction	- Small sample size<br>- No external validation
2021 (Zeng *et al.*)[7]	1. Cellular/tissue feature extraction using CellProfiler<br>2. Machine learning classifiers for prediction of molecular features<br>3. Multi-omics model for survival analysis	Patient n = 115<br>WSI n = unknown<br>(TCGA)	Patient n = 114<br>WSI n = unknown<br>(TCGA)	Patient n = 92<br>TMA core n = unknown<br>(single institution)	AUC = 0.703	5-year overall survival (OS) prediction	- Quantitative features were extracted
2022 (Boehm *et al.*)[8]	1. Cell-type feature extraction using QuPath<br>2. Resnet-18 CNN pretrained on ImageNet for tissue-type classification and extracting tissue-type features<br>3. Multimodal models integrated with histopathological, radiomic, genomic, and clinical data	Patient n = 243<br>WSI n = unknown<br>(TCGA and single institution)	Patient n = 40<br>WSI n = unknown<br>(with 4-fold cross-validation)<br>(TCGA and single institution)	Not done	C-Index = 0.54	OS prediction (regression)	- Histopathological image features were extracted<br>- No external validation
2022 (Wang *et al.*)[9]	1. Weakly supervised ROI sampling CNN based on FCN<br>2. Inception V3 CNN for tile-based predictions on probabilities in treatment effectiveness	Patient n = 187<br>WSI n = 187<br>(multi-institution)	Patient n = 101<br>WSI n = 101<br>(with 5-fold cross-validation)<br>(multi-institution)	Patient n = 71<br>TMA core n = 135<br>(institution unknown)	AUC = 0.933	Bevacizumab treatment response	- Limited to Bevacizimab treatment response<br>- TMA for external validation

CNN, convolutional neural network; PPV, positive predictive value; ROI, region of interest; TMA, tissue microarray
* No other performance metrics were provided.

References:

1. Yu, G., Wang, L.-G., Han, Y. & He, Q.-Y. clusterProfiler: an R Package for Comparing Biological Themes Among Gene Clusters. *OMICS J. Integr. Biol.* **16**, 284–287 (2012).
2. Telli, M. L. *et al.* Homologous recombination deficiency (HRD) status predicts response to standard neoadjuvant chemotherapy in patients with triple-negative or BRCA1/2 mutation-associated breast cancer. *Breast Cancer Res. Treat.* **168**, 625–630 (2018).
3. Takaya, H., Nakai, H., Takamatsu, S., Mandai, M. & Matsumura, N. Homologous recombination deficiency status-based classification of high-grade serous ovarian carcinoma. *Sci. Rep.* **10**, 2757 (2020).
4. Perez-Villatoro, F. *et al.* Optimized detection of homologous recombination deficiency improves the prediction of clinical outcomes in cancer. *Npj Precis. Oncol.* **6**, 1–13 (2022).
5. Yu, K.-H. *et al.* Deciphering serous ovarian carcinoma histopathology and platinum response by convolutional neural networks. *BMC Med.* **18**, 236 (2020).
6. Laury, A. R., Blom, S., Ropponen, T., Virtanen, A. & Carpén, O. M. Artificial intelligence-based image analysis can predict outcome in high-grade serous carcinoma via histology alone. *Sci. Rep.* **11**, 19165 (2021).
7. Zeng, H., Chen, L., Zhang, M., Luo, Y. & Ma, X. Integration of histopathological images and multi-dimensional omics analyses predicts molecular features and prognosis in high-grade serous ovarian cancer. *Gynecol. Oncol.* **163**, 171–180 (2021).
8. Boehm, K. M. *et al.* Multimodal data integration using machine learning improves risk stratification of high-grade serous ovarian cancer. *Nat. Cancer* **3**, 723–733 (2022).
9. Wang, C.-W. *et al.* Weakly supervised deep learning for prediction of treatment effectiveness on ovarian cancer from histopathology images. *Comput. Med. Imaging Graph.* **99**, 102093 (2022).
