# Supplementary material for: Histopathologic image–based deep learning classifier for predicting platinum-based treatment responses in high-grade serous ovarian cancer
Source: Nat Commun. 2024 May 18;15:4253. doi: 10.1038/s41467-024-48667-6 (PMC11102549; doi:10.1038/s41467-024-48667-6)
Supplement: Supplementary file 3 — Description of Additional Supplementary Files [file 41467_2024_48667_MOESM3_ESM.pdf]

Description of Additional Supplementary Files

Supplementary Data 1: Differentially expressed genes comparing the true and false favorable-predicted response groups.

Supplementary Data 2: Differentially expressed genes comparing the true and false poor-predicted response groups.

Supplementary Data 3: Differentially expressed genes comparing the ground truth favorable and poor response groups.
