# Supplementary material for: Histopathologic image–based deep learning classifier for predicting platinum-based treatment responses in high-grade serous ovarian cancer
Source: Nat Commun. 2024 May 18;15:4253. doi: 10.1038/s41467-024-48667-6 (PMC11102549; doi:10.1038/s41467-024-48667-6)
Supplement: Supplementary file 5 — Reporting Summary [file 41467_2024_48667_MOESM5_ESM.pdf]

nature portfolio

Corresponding author(s): Eunhyang Park

Last updated by author(s): May 3, 2024

Reporting Summary

Nature Portfolio wishes to improve the reproducibility of the work that we publish. This form provides structure for consistency and transparency in reporting. For further information on Nature Portfolio policies, see our Editorial Policies and the Editorial Policy Checklist.

Statistics

For all statistical analyses, confirm that the following items are present in the figure legend, table legend, main text, or Methods section.

n/a	Confirmed	
☐	☒	The exact sample size (*n*) for each experimental group/condition, given as a discrete number and unit of measurement
☒	☐	A statement on whether measurements were taken from distinct samples or whether the same sample was measured repeatedly
☐	☒	The statistical test(s) used AND whether they are one- or two-sided<br>*Only common tests should be described solely by name; describe more complex techniques in the Methods section.*
☐	☒	A description of all covariates tested
☒	☐	A description of any assumptions or corrections, such as tests of normality and adjustment for multiple comparisons
☐	☒	A full description of the statistical parameters including central tendency (e.g. means) or other basic estimates (e.g. regression coefficient) AND variation (e.g. standard deviation) or associated estimates of uncertainty (e.g. confidence intervals)
☐	☒	For null hypothesis testing, the test statistic (e.g. *F*, *t*, *r*) with confidence intervals, effect sizes, degrees of freedom and *P* value noted<br>*Give P values as exact values whenever suitable.*
☒	☐	For Bayesian analysis, information on the choice of priors and Markov chain Monte Carlo settings
☒	☐	For hierarchical and complex designs, identification of the appropriate level for tests and full reporting of outcomes
☒	☐	Estimates of effect sizes (e.g. Cohen's *d*, Pearson's *r*), indicating how they were calculated

Our web collection on statistics for biologists contains articles on many of the points above.

Software and code

Policy information about availability of computer code

Data collection	No software was used for data collection
Data analysis	We uploaded the open-source version of our model code in the repository [https://github.com/dmmoon/PathoRICH].<br>Additinal softwares for our data analyses are follows:<br>- GDCRNATools (version 1.16.6)<br>- clusterprofiler (version 4.4.4)<br>- IOBR package60 (version 0.99.9)<br>- R software (version 4.2.1)<br>- Sequencher 5.3 software<br>- Illumina BaseSpace software (Illumina)<br>- EnhancedVolcano package (version 1.14.0)

For manuscripts utilizing custom algorithms or software that are central to the research but not yet described in published literature, software must be made available to editors and reviewers. We strongly encourage code deposition in a community repository (e.g. GitHub). See the Nature Portfolio guidelines for submitting code & software for further information.

Data

Policy information about availability of data

All manuscripts must include a data availability statement. This statement should provide the following information, where applicable:

- Accession codes, unique identifiers, or web links for publicly available datasets
- A description of any restrictions on data availability
- For clinical datasets or third party data, please ensure that the statement adheres to our policy

Source data are provided as a Source Data file. The remaining data are available within the Article or Supplementary Data. The TCGA dataset is publicly available via the TCGA portal (https://portal.gdc.cancer.gov). The CAMELYON 16 and CAMELYON17 dataset is publicly available via Camelyon grand challenge website (https://camelyon16.grand-challenge.org, and https://camelyon17.grand-challenge.org respectively). SEV and SMC cohorts' NGS dataset for germline BRCA tests is deposited at following repository (https://www.ncbi.nlm.nih.gov/sra/PRJNA1108881). WSI data for the SEV and SMC cohorts are not publicly available due to hospital regulations. The data could be available on request from the corresponding author (E.P.) and response will be received typically within 4 weeks. Data usage is restricted to non-commercial academic research purpose.

Research involving human participants, their data, or biological material

Policy information about studies with human participants or human data. See also policy information about sex, gender (identity/presentation), and sexual orientation and race, ethnicity and racism.

Reporting on sex and gender	This study was conducted on female patients diagnosed with high-grade serous ovarian carcinoma. All patients were biologically confirmed as female through clinical examinations.
Reporting on race, ethnicity, or other socially relevant groupings	Our research, which aims to predict treatment outcomes in high-grade serous ovarian carcinoma, is based solely on the analysis of histological images. It does not involve or require the use of variables such as race, ethnicity, or other social categories. Therefore, our study did not account for or control confounding variables related to such categories, as they were not relevant to our image-based predictive model.
Population characteristics	The patient population in our study comprises female high-grade serous ovarian carcinoma patients who underwent primary debulking surgery followed by adjuvant platinum-based chemotherapy. Specific to the internal SEV cohort and external SMC cohort, patients did not receive PARP inhibitors within the first two years post-diagnosis.
Recruitment	Patients for our study were retrospectively selected by examining medical records and pathology reports. Pathologists reviewed whole slide images (Wls) trom debulking surgeries to select representative slides. While slides with artifacts, poor staining quality, or insufficient cancerous regions were excluded, lack of stadnardized selection criteria for the WSIs could have introduced selection bias, arising from the subjective decisions of the pathologists.
Ethics oversight	This study was approved by the institutional review board of Severance Hospital (IRB no. 4-2021-1391). Informed consent was waived for this retrospective study and participants were not compensated.

Note that full information on the approval of the study protocol must also be provided in the manuscript.

Field-specific reporting

Please select the one below that is the best fit for your research. If you are not sure, read the appropriate sections before making your selection.

☒ Life sciences ☐ Behavioural & social sciences ☐ Ecological, evolutionary & environmental sciences

For a reference copy of the document with all sections, see nature.com/documents/nr-reporting-summary-flat.pdf

Life sciences study design

All studies must disclose on these points even when the disclosure is negative.

Sample size	The study's sample size was determined by examining medical records and pathology reports to identify patients diagnosed with high-grade serous ovarian carcinoma who underwent debulking surgery. We then further narrowed our focus to those who received adjuvant chemotherapy following their surgery for inclusion in the study.
Data exclusions	In our study, we excluded patients from the internal SEV and external SMC cohorts who did not complete at least six cycles of adjuvant platinum-based chemotherapy. This exclusion criterion was applied to ensure that the patients had received adequate adjuvant treatment, which is critical for the validity of our model aiming to predict the response to platinum-based chemotherapy using only histological imagery.
Replication	To ensure the reproducibility of our experimental findings, we utilized a 5-fold cross-validation technique within the SEV cohort for training and validation purposes. Furthermore, we used two additional external cohorts, TCGA and SMC, to further assess and validate the robustness and generalizability of our predictive model.
Randomization	In the SEV cohort, we implemented 5-fold cross-validation where the samples were randomized during the process.

Blinding	Blinding was not relevant to our study design as the analysis was based solely on the objective assessment of histological images using computational models. There was no group allocation that required blinding during data collection or analysis.

Reporting for specific materials, systems and methods

We require information from authors about some types of materials, experimental systems and methods used in many studies. Here, indicate whether each material, system or method listed is relevant to your study. If you are not sure if a list item applies to your research, read the appropriate section before selecting a response.

Materials & experimental systems

n/a	Involved in the study
☒	☐ Antibodies
☒	☐ Eukaryotic cell lines
☒	☐ Palaeontology and archaeology
☒	☐ Animals and other organisms
☒	☐ Clinical data
☒	☐ Dual use research of concern
☒	☐ Plants

Methods

n/a	Involved in the study
☒	☐ ChIP-seq
☒	☐ Flow cytometry
☒	☐ MRI-based neuroimaging
